# Integrating Novel Biomarkers into Clinical Practice: A Practical Framework for Diagnosis and Management of Cardiorenal Syndrome

**DOI:** 10.3390/life15101540

**Published:** 2025-10-01

**Authors:** Georgios Aletras, Maria Bachlitzanaki, Maria Stratinaki, Emmanuel Lamprogiannakis, Ioannis Petrakis, Emmanuel Foukarakis, Yannis Pantazis, Michael Hamilos, Kostas Stylianou

**Affiliations:** 1 Department of Cardiology, Venizelio General Hospital of Heraklion, 71409 Heraklion, Greece; medp2012222@med.uoc.gr (G.A.); maria.stratinaki@gmail.com (M.S.); mlamprog@yahoo.com (E.L.); mfouk@hotmail.com (E.F.); 2 School of Medicine, University of Crete, 70013 Heraklion, Greece; medp2011922@med.uoc.gr (M.B.); petrakgia@uoc.gr (I.P.); mchamilos@uoc.gr (M.H.); 3 Second Department of Internal Medicine, Venizelio General Hospital of Heraklion, 71409 Heraklion, Greece; 4 Department of Nephrology, University General Hospital of Heraklion, 71500 Heraklion, Greece; 5 Institution of Applied and Computational Mathematics, Foundation of Research and Technology-Hellas, 70013 Heraklion, Greece; pantazis@iacm.forth.gr; 6 Department of Cardiology, University General Hospital of Heraklion, 71500 Heraklion, Greece

**Keywords:** cardiorenal syndrome, novel biomarkers, tubular injury, inflammation, fibrosis, clinical practice

## Abstract

Cardiorenal syndrome (CRS) reflects the intricate and bidirectional interplay between cardiac and renal dysfunction, commonly resulting in diagnostic uncertainty, therapeutic dilemmas and poor outcomes. While traditional biomarkers like serum creatinine (Cr) and natriuretic peptides remain widely used, their limitations in specificity, timing and contextual interpretation often hinder optimal management. This narrative review synthesizes the current evidence on established and emerging biomarkers in CRS, with emphasis on their clinical relevance, integration into real-world practice, and potential to inform precision therapy. Markers of glomerular filtration rate beyond creatinine—such as cystatin C—offer more accurate assessment in frail or sarcopenic patients, while tubular injury markers such as NGAL, KIM-1, and urinary L-FABP (uL-FABP) provide early signals of structural renal damage. The FDA-approved NephroCheck^®^ test—based on TIMP-2 and IGFBP7— enables risk stratification for imminent AKI up to 24 h before functional decline. Congestion-related markers such as CA125 and bio-adrenomedullin outperform natriuretic peptides in certain CRS phenotypes, particularly in right-sided heart failure or renally impaired patients. Fibrosis and inflammation markers (galectin-3, sST2, GDF-15) add prognostic insights, especially when combined with NT-proBNP or troponin. Rather than presenting biomarkers in isolation, this review proposes a framework that links them to specific clinical contexts—such as suspected decongestion-related renal worsening or persistent congestion despite therapy—to support actionable interpretation. A tailored, scenario-based, multi-marker strategy may enhance diagnostic precision and treatment safety in CRS. Future research should prioritize prospective biomarker-guided trials and standardized pathways for clinical integration.

## 1. Introduction

Heart failure (HF) and chronic kidney disease (CKD) are closely interconnected conditions that share a wide array of pathophysiological mechanisms, risk factors, and clinical features. Both are characterized by neurohormonal activation, endothelial dysfunction, inflammation, and fibrotic remodeling, and they often respond to the same pharmacological interventions [1,2]. Indeed, several therapeutic classes—including renin–angiotensin–aldosterone system inhibitors (RAASis), non-steroidal mineralocorticoid receptor antagonists (ns-MRAs), sodium-glucose cotransporter-2 inhibitors (SGLT2is), and glucagone-like-peptide-1 receptor agonists (GLP-1 RAs)—have demonstrated benefits in both HF and CKD, underscoring the overlap in their pathogenesis and treatment strategies [3,4,5,6].

From an epidemiological perspective, CKD affects approximately 844 million individuals globally, while HF affects around 64 million [7,8]. Notably, CKD coexists in nearly 50% of patients with HF, and the coexistence of both conditions confers a particularly poor prognosis [8,9]. It is now well established that the incidence of HF is significantly higher among individuals with CKD, while HF itself accelerates renal decline, particularly through hemodynamic alterations, venous congestion, and neurohormonal dysregulation [10].

An additional challenge is the asymptomatic nature of early-stage CKD, which often leads to delayed diagnosis at more advanced stages, when substantial nephron loss has already occurred. In a landmark study by Dalrymple et al. (2011), patients aged over 65 years with CKD were found to be six times more likely to die from cardiovascular causes than to progress to end-stage renal disease (ESRD), illustrating that most patients with progressive CKD die before reaching kidney failure [11,12].

These complex, bidirectional interactions between the heart and kidneys have led to the conceptualization of cardiorenal syndrome (CRS)—a term first officially defined by the National Heart, Lung, and Blood Institute in 2004 [13]. CRS encompasses acute or chronic dysfunction in the heart or kidneys that induces dysfunction in the other organ. It is currently classified into five subtypes based on the primary failing organ (heart vs. kidney), the chronicity (acute versus chronic), and whether the dysfunction is secondary to a systemic condition affecting both organs (Appendix A) [1,14,15]. Although this classification was intended to aid in diagnosis and therapeutic decision-making, in reality, it offers limited practical value. While it helps conceptually organize the different pathways of heart–kidney interaction, clinical presentations are often more complex, with overlapping features and dynamic transitions between acute and chronic states that do not fit neatly into predefined categories [13].

More recently, the concept of cardiovascular–kidney–metabolic (CKM) syndrome has emerged to capture the shared metabolic derangements and systemic drivers that link these conditions. This broader framework recognizes the interconnected nature of cardiovascular, renal, and metabolic health, and highlights the need for integrated diagnostic and therapeutic strategies [16].

In this evolving landscape, there is a growing need for tools that can more accurately reflect the complexity and heterogeneity of cardiorenal interactions. Traditional markers such as serum creatinine, estimated glomerular filtration rate (GFR), and natriuretic peptides (NPs) provide important information but have well-recognized limitations, particularly in the early detection, risk stratification, and monitoring of CRS. As our understanding of the pathophysiology deepens, a range of novel biomarkers has emerged, offering the potential to better characterize disease trajectories, identify sub-phenotypes, and guide individualized therapy. This narrative review will explore the most recent advances in biomarker research within the context of CRS, with a focus on their clinical relevance and future applications.

## 2. Literature Review Strategy

The literature review was conducted using a narrative approach focused on clinically relevant biomarkers in the context of CRS. Searches were performed in PubMed, Scopus, and Web of Science using combinations of keywords such as “cardiorenal syndrome,” “biomarkers,” “heart failure,” “acute kidney injury,” “chronic kidney disease,” and specific marker names (e.g., NGAL, KIM-1, cystatin C, CA125, bio-ADM, FGF-23, etc.).

Priority was given to:

○ Original research, systematic reviews, and meta-analyses published in the past 10–15 years
○ Landmark cohort studies, randomized controlled trials, and FDA-related regulatory approvals
○ Publications in high-impact cardiology and nephrology journals

References were selected based on clinical applicability, study quality, and relevance to the CRS framework. Where available, we extracted and contextualized cut-off values, assay types, and prognostic data to support practical implementation. Special emphasis was placed on biomarker interpretation in dynamic clinical settings, including decongestion, worsening renal function (WRF) assessment, and acute kidney injury (AKI) risk stratification.

## 3. Beyond Creatinine and Natriuretic Peptides: Gaps in Cardiorenal Assessment

### 3.1. Limitations of Creatinine and Estimated Glomerular Filtration Rate

The gold standard of measuring GFR is through exogenous markers such as iothalamate or inulin, as these molecules are freely filtered and largely without renal secretion or/and absorption. However, this is cumbersome and impractical for routine use in clinical practice [17]. In routine clinical practice, renal function is almost exclusively assessed by serum creatinine (Cr) and Cr-based estimations of the GFR [18].

Creatinine is a product of muscle catabolism that is freely filtered by the glomerulus, with additional minor tubular secretion. It is cheap and easy to measure. While the use of creatinine-based GFR estimates has been instrumental in defining kidney disease as a global health problem, creatinine is not always the ideal surrogate marker for GFR [19]. This approach has well-known limitations such as Cr analytic assay variability, Cr secretion by the proximal renal tubules (rendering it an imperfect biomarker for glomerular filtration), as well as dependency of serum Cr levels on muscle mass, age, sex, diet, and physical activity [18]. Moreover, serum Cr is a relatively late and insensitive marker of AKI, often increasing only after substantial loss of kidney function has occurred, when early recognition of AKI is crucial to allow timely intervention [20]. Serum Cr levels may also transiently increase in HF, reflecting hemodynamic changes rather than structural injury [17,18]. Observational studies also have shown that patients discharged with unresolved congestion, despite modest Cr rises, have worse outcomes than those fully decongested, while the same principle applies in the initiation/uptitration of neurohormonal blockers. Therefore, Cr and eGFR must be interpreted in clinical context, alongside markers of congestion and tubular injury [19,21,22].

### 3.2. Nephron Dynamics and Renal Reserve

Mechanistically, GFR is the product of the number of functional nephrons and the single-nephron GFR (SNGFR). Each kidney is endowed with approximately one million nephrons at birth. This large nephron pool allows for substantial renal reserve capacity, enabling compensation for nephron loss through an increase in SNGFR, thereby preserving total GFR during the early phases of nephron reduction. Total GFR thus represents a dynamic equilibrium between nephron quantity and individual nephron function, modulated by systemic and intraglomerular hemodynamics as well as neurohormonal influences [17,18]. In contrast, the number of viable nephrons declines progressively with age—estimated at 5000–10,000 per year—resulting in a gradual depletion of renal reserve and, eventually a decline in overall GFR [18,23]. Although compensatory hyperfiltration at the single-nephron level may initially maintain GFR, this adaptive response is finite and, over time, contributes to glomerular damage, podocyte loss and the progression of chronic kidney disease [24].

In its purest sense, CKD is defined as a greater loss of functional nephrons than would be anticipated through healthy aging. A GFR < 60 mL/min/1.73 m^2^ is therefore used as a reasonable surrogate, with this cutoff corresponding to a >50% loss of functional nephrons at a normal single-nephron GFR. Importantly, an increased single-nephron GFR may compensate for a loss of functional nephrons, reducing the impact on the total GFR assessed by serum Cr, whereas albuminuria is a distinct measure reflecting glomerular and tubular damage and/or function [18,25]. The hypothesis is that in such patients, increased urinary albumin (≥30 mg/g Cr) reflects an increased single-nephron GFR (i.e., glomerular hyper-filtration) and, consequently, a lower number of functional nephrons than reflected by the total GFR, such as in diabetic kidney disease. Moreover, glomerular hyper-filtration is a key pathophysiological mechanism underlying further CKD progression, as it results in accelerated podocyte loss and a dysfunctional glomerular basement membrane. Albuminuria and its implications will be analyzed in a specific section [18,25].

### 3.3. Limitations of Natriuretic Peptides and Troponins

In parallel, natriuretic peptides, particularly NT-proBNP, and cardiac troponins, such as high-sensitivity cardiac troponin I (hs-cTnI), are commonly employed for risk stratification and diagnosis in HF, yet their utility in capturing the complexity of CRS is limited. NT-proBNP reflects myocardial wall stress and volume overload, but its levels are influenced by age, obesity, and renal function [15]. In patients with CKD or CRS, elevated NT-proBNP may not reliably differentiate between cardiac congestion and renal impairment, as impaired clearance contributes to persistently high levels regardless of actual hemodynamic status [26]. Similarly, hs-cTnI is a sensitive marker of myocardial injury and is often elevated in CRS due to subclinical myocardial stress, but it lacks specificity for reversible versus irreversible injury, and its interpretation can be challenging in the context of coexisting renal dysfunction. Thus, while both biomarkers offer prognostic information, they have limitations in guiding therapy in CRS [15].

## 4. A Primer on Novel Biomarker Classes in CRS

### 4.1. Glomerular Filtration Markers Beyond Creatinine

#### 4.1.1. Cystatin-C

Cystatin C is a low-molecular-weight cysteine protease inhibitor produced by all nucleated cells at a relatively constant rate. It is freely filtered by the glomerulus and almost completely reabsorbed and metabolized by proximal tubular epithelial cells, making it an attractive endogenous marker of GFR [27]. Unlike serum creatinine, cystatin C is minimally influenced by muscle mass, age, or nutritional status, which enhances its reliability in patients with sarcopenia, cachexia, or other conditions where creatinine-based estimates may be misleading [15,17,28].

Because of its stability and reduced dependence on non-GFR factors, cystatin C has gained traction as a marker for cardiovascular (CV) and renal risk stratification. Elevated cystatin C levels have been associated with worse outcomes in a range of clinical scenarios, including coronary artery disease (CAD), acute and chronic HF, and chronic kidney disease [15,29]. In patients hospitalized for acute decompensated HF (ADHF), serum cystatin C levels at admission were found to be superior to both serum creatinine and B-type natriuretic peptide (BNP) in predicting all-cause mortality and rehospitalization [30].

Nevertheless, cystatin C is not without limitations. Its levels can be affected by several non-GFR determinants, including thyroid dysfunction, obesity, systemic inflammation, and corticosteroid use [15,17]. A recent study in 293 HF patients found that cystatin C levels, although less affected by muscle mass than creatinine, still showed some correlation with it—potentially reflecting underlying metabolic and inflammatory processes rather than direct muscle mass influence [31].

One of the most compelling insights into the utility of cystatin C in HF comes from population-based studies. In a large real-world cohort from Sweden, involving over 158.000 individuals, discordance between eGFR_Cr_ and eGFR_Cys_ was common and clinically meaningful. In 65% of cases, eGFR_Cys_ was lower than eGFR_Cr_, with over a quarter of patients showing a difference exceeding 27%. This discrepancy was most pronounced in older individuals with multiple comorbidities—a profile commonly seen in HF populations—and was independently associated with higher risk of acute kidney injury (AKI), progression to kidney failure, HF events, and all-cause mortality [32]. These findings underscore the potential of cystatin-C to uncover hidden renal dysfunction in patients where creatinine may overestimate true GFR [33].

The importance of using cystatin C in conjunction with creatinine was further highlighted in a subanalysis of the PARADIGM-HF trial. When comparing the 2021 CKD-EPI combined creatinine–cystatin C equation to the creatinine-only equation, substantial individual reclassification occurred, particularly in sicker patients. A lower GFR estimated by the combined equation was associated with higher levels of natriuretic peptides and troponin, poorer quality of life as assessed by the Kansas City Cardiomyopathy Questionnaire (KCCQ), and a higher risk of mortality. These discrepancies were likely due to a less pronounced increase in creatinine compared to cystatin C in patients with greater neurohormonal activation—possibly as a result of decreased creatinine production or increased tubular secretion in advanced HF [34].

In summary, while cystatin C is not entirely exempt from confounding factors, it remains a more sensitive and less muscle-mass-dependent marker of GFR compared to serum Cr. It may enhance early detection of renal impairment, refine risk stratification, and guide clinical decisions in heart failure—particularly in frail, elderly, or cachectic individuals. Furthermore, the observed discordance between creatinine and cystatin C-based eGFR may serve as an indirect marker of frailty and worse prognosis, reinforcing the clinical value of incorporating cystatin C into routine assessment of patients with CRS [33] (Table 1).

#### 4.1.2. Βeta-2-Microglobulin (β2-M)

Beta-2-microglobulin (β2M) is a low-molecular-weight protein produced by all nucleated cells, continuously released into the circulation, freely filtered by the glomerulus, and almost entirely reabsorbed and metabolized by proximal tubular cells, making it a useful marker of GFR [36].

Serum β2M levels rise progressively with declining GFR, and its kinetics—similar to cystatin C—support its use as an alternative marker of glomerular filtration, particularly where creatinine-based estimates are less reliable. Unlike creatinine, β2M is not secreted by the renal tubules, minimizing potential biases in GFR estimation, and it does not appear to be influenced by sex [37]. However, despite these theoretical benefits, β2M concentrations can still be influenced by non-renal factors, including inflammation, malignancy and autoimmune diseases—all of which increase β2M production and turnover [36].

From a laboratory perspective, β2M is typically measured using immunoassays, though standardization is less advanced compared with cystatin C. The marker is generally stable in stored serum and plasma, but pre-analytical factors such as hemolysis or systemic inflammation may influence results. Furthermore, at higher GFR levels, β2M may also underestimate renal function, likely related to variability in extra-renal production and clearance pathways. These limitations mean that while β2M is a valid GFR marker, cystatin C often remains the preferred choice for routine estimation of kidney function beyond creatinine [36,38].

In addition to its filtration marker role, serum β2M has prognostic relevance. It is strongly associated with all-cause mortality, cardiovascular events, and progression to end-stage renal disease (ESRD) in both CKD populations and healthy individuals [36,39,40,41,42]. For example, in the CRIC Study, higher serum β2M independently predicted ESRD, mortality, and incident cardiovascular disease, occasionally outperforming creatinine-based eGFR [40]. In dialysis patients, β2M levels reflect residual renal function and correlate with cardiovascular outcomes, although they are also influenced by dialytic clearance efficiency, underlying inflammation, and the risk of dialysis-related β2M amyloidosis—a condition characterized by β2M fibril deposition with osteoarticular, dermatologic, gastrointestinal, and cardiovascular manifestations [36,43].

Urinary β2M, historically used to assess proximal tubular injury, is less commonly employed today due to its instability in acidic urine, limited specificity for tubular over glomerular injury and modest predictive performance [36].

In summary, β2M is a valuable alternative filtration marker and a potent prognostic biomarker in CKD and CRS. However, its clinical application is constrained by non-renal influences and greater familiarity with cystatin C in clinical settings. The combined assessment of β2M with other filtration markers may enhance risk stratification, particularly where creatinine or cystatin C measurements are ambiguous or less informative [38,41].

#### 4.1.3. Proenkephalin (PENK)

Proenkephalin (PENK) is an endogenous opioid peptide and a stable surrogate of active enkephalins, synthesized primarily in the central nervous system. In the kidney, enkephalins modulate renal perfusion, diuresis, and natriuresis by activating delta-opioid receptors, especially in the proximal tubules, where receptor density is high [44,45]. PENK itself is freely filtered at the glomerulus and is not significantly influenced by age or sex in most cohorts, making it a promising marker of GFR. Studies have consistently shown a strong correlation between PENK levels and measured GFR across diverse populations, including those with CKD, HF, and post-cardiac surgery patients [45,46].

Beyond reflecting renal filtration, PENK levels may rise as a counterregulatory response to renal hypoperfusion or oxidative stress, conditions common in HF and CRS [45,47]. Its concentration has been associated with the risk of AKI in critically ill patients and may outperform traditional markers in early AKI detection [48,49]. Furthermore, elevated PENK levels have been linked to adverse outcomes in ADHF, including worsening renal function, rehospitalization, and all-cause mortality. Notably, PENK levels also correlate with other renal biomarkers such as NGAL, indicating its relevance in tubular stress contexts. Most studies quantify PENK using sandwich immunoassays, but no universally accepted clinical cut-off has been established, and reference ranges remain assay- and laboratory-specific [47,48,50,51].

While PENK tracks measured GFR across diverse cohorts, elevations in advanced HF may also reflect neurohormonal activation and systemic stress, limiting its specificity as a pure filtration surrogate. There is also variability in cutoff thresholds across studies, and its utility in guiding therapy remains unproven. While promising for risk stratification in CRS and acute HF, further research is needed to clarify its prognostic value and define standardized reference ranges [44,45,46,48].

### 4.2. Markers of Tubular Injury

#### 4.2.1. Albuminuria

Albumin is a small, negatively charged plasma protein that plays a key role in maintaining oncotic pressure and transporting endogenous and exogenous molecules. Under normal conditions, the glomerular filtration barrier—composed of fenestrated endothelial cells, the glomerular basement membrane (GBM), and interdigitating podocytes connected by slit diaphragms—effectively prevents the passage of albumin into the urine. Disruption of this barrier leads to increased urinary albumin excretion, a phenomenon known as albuminuria [25,52]. Clinically, albuminuria is defined by a urinary albumin-to-creatinine ratio (UACR) ≥ 30 mg/g and is categorized into moderately increased-A2 (30–300 mg/g) and severely increased-A3 (>300 mg/g) levels [5]. Among patients with chronic HF, elevated UACR is observed in approximately 25% to 44% of cases, highlighting its high prevalence in this population [18,53,54].

Although albuminuria is traditionally considered a marker of glomerular barrier dysfunction, proximal tubular abnormalities may also contribute to increased urinary albumin excretion in specific clinical contexts. For example, in inherited Fanconi syndrome or drug-induced nephrotoxicity (e.g., Tenofovir, Aminoglycosides, Cisplatin), albuminuria may arise due to impaired tubular reabsorption [55]. Additionally, in diabetes mellitus, early dysfunction of megalin–cubilin receptor-mediated endocytosis in proximal tubules may reduce albumin reuptake, contributing to microalbuminuria even before overt glomerular damage. Thus, albuminuria may in some cases reflect early tubular dysfunction in addition to glomerular injury, especially in diseases characterized by complex nephron involvement [56].

Beyond its role as a marker of glomerular and proximal tubular damage, albuminuria reflects broader systemic processes involved in cardiovascular disease (CVD). The pathophysiological mechanisms linking albuminuria to HF are multifactorial, encompassing glomerular injury, systemic endothelial dysfunction, low-grade inflammation, neurohormonal activation—particularly of the renin–angiotensin–aldosterone system (RAAS) causing directly podocyte injury—and elevated central venous pressure leading to renal congestion [57,58].

Albuminuria is prevalent in both HF with reduced ejection fraction (HFrEF) and preserved ejection fraction (HFpEF), and is associated with adverse outcomes regardless of eGFR. Even mild elevations within the traditionally “normal” range (<30 mg/g) have been associated with an increased risk of heart failure and adverse cardiovascular outcomes, reinforcing the role of albuminuria as a sensitive marker of both early and progressive cardiorenal dysfunction [25,59]. Albuminuria thus serves not only as a marker of glomerular injury but also as an independent prognostic indicator in CRS [25,57]. Notably, albuminuria frequently occurs even in patients without diabetes or overt renal impairment, underscoring its utility as an early indicator of cardiorenal stress. Cohort data, including these from the Framingham Heart Study, CHARM and CHART-2 trials, have shown that even microalbuminuria (30–300 mg/g) is associated with increased risk of incident HF, hospitalization, and mortality [10,53,59,60,61]. In HFpEF populations specifically, such as those studied in TOPCAT, albuminuria was present in up to one-third of patients and independently predicted HF hospital readmission [62].

Recent evidence also suggests that albuminuria may reflect systemic congestion rather than intrinsic kidney damage alone. In HF cohorts, elevated UACR has been closely associated with markers of volume overload such as NT-proBNP, bio-adrenomedullin, peripheral edema, and echocardiographic signs of increased right-sided pressures. These associations persist independently of GFR and tubular injury markers, suggesting that albuminuria may also reflect venous congestion and systemic endothelial dysfunction in CRS, rather than intrinsic renal injury alone [58]. Levels often improve with decongestion [62], and this therapeutic responsiveness can help distinguish congestion-driven albuminuria from intrinsic nephropathy [63].

#### 4.2.2. Neutrophil Gelatinase-Associated Lipocalin (NGAL)

Neutrophil Gelatinase-Associated Lipocalin (NGAL), also known as lipocalin-2, is a 25-kDa glycoprotein primarily secreted by activated neutrophils and also expressed in a wide array of tissues, including renal tubular cells, cardiomyocytes, hepatocytes, and adipocytes. It is involved in iron transport, innate immunity, and cellular stress responses [64].

NGAL is one of the most extensively studied biomarkers for AKI, especially due to its rapid release following tubular injury [20]. Transcriptomic studies in animal models have shown that NGAL is among the earliest genes induced after kidney insult, particularly in distal nephron segments [65,66]. In both plasma and urine, NGAL levels rise within 2–6 h of renal injury, well before serum creatinine increases, thus enabling earlier detection of AKI [64]. Commercial assays for NGAL have been approved in several regions for AKI detection, and its clinical utility is being increasingly recognized in the context of CRS. Assay platforms include chemiluminescent microparticle immunoassay (CMIA), ELISA, and point-of-care devices, though availability and regulatory approval vary across regions [20,64].

In patients with HF, elevated NGAL levels—both plasma and urinary—have been consistently associated with impaired renal function, increased NT-proBNP, urinary albumin excretion, and higher mortality risk [67,68,69]. NGAL is particularly useful in identifying patients at risk of developing CRS type 1, in which acute HF precipitates AKI. Studies have shown that NGAL levels at admission can predict subsequent AKI in acute decompensated HF and correlate with worsening renal function and clinical outcomes [64,70,71,72,73]. In CRS type 2, NGAL may also contribute to pathogenesis through modulation of matrix metalloproteinase-9 (MMP-9) activity and extracellular matrix remodeling. This interaction promotes extracellular matrix degradation and remodeling, processes that are implicated in renal fibrosis and progression of CKD [64,74].

While NGAL is a highly sensitive biomarker, its expression is also upregulated in response to epithelial injury, inflammation, and ischemia. This broad responsiveness—although useful in signaling global pathophysiological stress—reduces specificity for renal tubular origin. Plasma NGAL may be particularly affected by systemic inflammation, anemia and sepsis, whereas urinary NGAL appears more specific for tubular injury in HF/CRS populations [15,20,75]. Therefore, clinical interpretation of NGAL levels requires careful consideration of the broader clinical context, and combining NGAL with complementary markers (e.g., NT-proBNP, C-reactive protein, etc.) may enhance diagnostic precision [15].

#### 4.2.3. Kidney Injury Molecule-1 (KIM-1)

Kidney Injury Molecule-1 (KIM-1), also known as T-cell immunoglobulin mucin receptor 1 (TIM-1), is a 38.7-kDa type I transmembrane glycoprotein primarily expressed on the apical surface of proximal tubular epithelial cells. In healthy kidneys, KIM-1 is barely detectable, but its expression increases significantly after ischemic or toxic injury [76,77]. KIM-1 may also be present in other tissues such as the intestine and biliary system, but its expression in these organs is markedly lower compared to the kidney [76]. Following renal injury, KIM-1 is shed into the urine and blood, where it can be measured as a non-invasive biomarker [77,78]. Beyond its renal role, KIM-1 participates in immunoregulation through interactions with T-cell subsets and may exert anti-inflammatory and phagocytic functions during epithelial injury [76,79].

KIM-1 is one of the most promising markers of proximal tubular injury, particularly in the early stages of acute kidney injury AKI [80,81]. It is rapidly upregulated after ischemia–reperfusion injury and may precede histological changes [82]. Urinary KIM-1 has shown diagnostic and prognostic value in multiple settings, including post-cardiac surgery AKI, diabetic nephropathy, kidney transplant rejection, and nephrotoxic drug exposure (aminoglycosides, non-steroidal anti-inflammatory drugs, polymyxin etc.) [15,78,80]. In patients with HF, elevated urinary KIM-1 levels have been associated with WRF and increased mortality risk. Some studies demonstrated that urinary KIM-1 could identify HF patients at high risk of post-discharge mortality and CRS progression [69,83].

Despite its high specificity, its sensitivity for detecting AKI is moderate, and diagnostic accuracy may be influenced by comorbidities, proteinuria, and inflammatory conditions. Additionally, discrepancies between urinary and plasma KIM-1 levels complicate interpretation. In clinical practice, urinary KIM-1 is generally preferred for early tubular injury screening, whereas plasma KIM-1 should be interpreted with caution in acute decompensated HF (ADHF) until further validation is available [78,84,85,86].

#### 4.2.4. Liver-Type Fatty Acid Binding Protein (L-FABP)

Liver-type fatty acid binding protein (L-FABP) is a 14-kDa cytoplasmic protein primarily expressed in the proximal tubular epithelial cells of the kidney. Under physiological conditions, L-FABP facilitates the intracellular transport and metabolism of long-chain fatty acids, protecting the tubular cells from lipotoxicity. Its expression is upregulated in response to hypoxia, oxidative stress, and ischemia—key mechanisms underlying both acute and chronic kidney injury [87,88].

In the setting of renal ischemia–reperfusion, tubular injury, or proteinuric kidney disease, L-FABP is released into the urine (uL-FABP), where it serves as a sensitive marker of early tubular stress. Unlike many other markers that reflect glomerular injury, uL-FABP is more closely associated with tubular dysfunction, offering unique insight into the pathophysiological processes of the renal tubulointerstitium [89,90]. In animal and human studies, urinary L-FABP levels have been shown to correlate with the severity of tubular damage and fibrotic changes, even in the absence of overt proteinuria [90].

From a clinical perspective, uL-FABP has demonstrated utility in both acute and chronic settings. It has been validated as an early marker of AKI, particularly in perioperative states, contrast-induced nephropathy, and critically ill populations. In patients with ADHF, elevated urinary L-FABP levels are predictive of AKI development and are associated with worse prognosis [90]. Several studies have shown that patients with higher L-FABP levels at admission were more likely to experience AKI, all-cause mortality, or adverse renal outcomes. In this context, L-FABP reflects underlying tubular stress that may not yet be apparent through traditional markers like serum Cr [89,91].

From a laboratory perspective, urinary L-FABP requires prompt sample processing or stabilization, as the protein is prone to degradation if stored at room temperature. In clinical studies, results are often reported by tertiles or quartiles of risk rather than universal cut-offs, since threshold values vary substantially depending on the assay platform [92,93].

In CKD, L-FABP has also shown promise as a predictor of disease progression, particularly in individuals with diabetic nephropathy or cardiovascular comorbidities. Importantly, uL-FABP may be especially helpful in stratifying risk among CKD patients with little or no albuminuria, where traditional markers may underestimate renal damage. Its role in reflecting ongoing oxidative stress and lipid-mediated tubular injury supports its use in early intervention strategies and long-term monitoring [88,90,94,95,96,97].

Moreover, the combination of L-FABP with established cardiac biomarkers such as NT-proBNP has been proposed as a powerful tool for identifying patients at risk of CRS. Studies in cardiac intensive care unit populations have shown that patients with elevations in both biomarkers were several times more likely to develop AKI than those with lower levels, suggesting additive predictive value and pathophysiological complementarity [98].

Despite its growing potential, certain limitations remain. While uL-FABP is sensitive and correlates well with tubular injury, its role as a monitoring tool during therapy remains to be fully elucidated. Furthermore, large-scale validation in diverse HF and CKD populations is still needed. Nevertheless, current evidence supports the integration of L-FABP into cardiorenal risk assessment, particularly in settings where subclinical kidney injury precedes overt declines in glomerular filtration [89,90,98].

#### 4.2.5. Tissue Inhibitor of Metalloproteinases-2 (TIMP-2) and Insulin-like Growth Factor Binding Protein 7 (IGFBP7): Early Markers of Tubular Stress

Tissue inhibitor of metalloproteinases-2 (TIMP-2) and insulin-like growth factor-binding protein 7 (IGFBP7) are markers of early distal and proximal tubular stress respectively. Both are involved in G1 cell cycle arrest, a protective mechanism that prevents cell division during injury. Unlike traditional markers, they do not reflect established injury but rather cellular stress preceding overt tubular damage [99]. Combined as [TIMP-2] × [IGFBP7], these biomarkers form the basis of the NephroCheck^®^ test, which has been Food and Drug Administration (FDA)-cleared for assessing the risk of moderate to severe AKI (KDIGO stage 2–3) within 12–24 h in ICU and post-cardiac surgery settings (Table 2). Their elevation prompts early preventive strategies, including optimization of hemodynamics, avoidance of nephrotoxins, and enhanced monitoring [100,101,102,103]. In clinical studies, this combination has shown strong predictive performance in high-risk populations, including patients with sepsis, shock, and those undergoing major surgery or kidney transplantation. Elevated [TIMP-2] × [IGFBP7] levels are also associated with prolonged delayed graft function and worse prognosis in transplant recipients [99].

While they are not substitutes for serum creatinine or urine output, they offer a valuable early warning signal—enabling proactive interventions before structural kidney damage becomes evident. Their role is particularly relevant in critically ill or hemodynamically unstable patients, and expanding use in pediatric and neonatal populations has also been reported [101,103,104,105].

### 4.3. Markers of Inflammation and Fibrosis

#### 4.3.1. Interleukin-18

Interleukin-18 (IL-18) is a pro-inflammatory cytokine from the interleukin-1 (IL-1) family, structurally and functionally similar to IL-1β. It is produced in an inactive form by various cell types, including monocytes, macrophages, and renal tubular epithelial cells, and becomes biologically active after cleavage by caspase-1. IL-18 plays a central role in immune regulation by promoting interferon-gamma (IFN-γ) release and driving a Th1-type immune response through the myeloid-differentiation factor 88(MyD88)/nuclear factor kappa B (NF-κB) signaling pathway. Under normal conditions, its activity is modulated by IL-18 binding protein (IL-18BP), but in many inflammatory diseases, this balance is disrupted, leading to excessive IL-18 activity [106,107].

Beyond its role in immune regulation, IL-18 has been implicated in the pathogenesis of cardiovascular disease. Higher circulating IL-18 levels have been linked to an increased risk of coronary artery disease, acute myocardial infarction, and the development or progression of HF. Several large cohort studies and meta-analyses have confirmed its association with adverse cardiovascular outcomes and mortality, even in the general population [108].

In the kidney, IL-18 is predominantly released from proximal tubular cells in response to ischemic or inflammatory injury. Urinary IL-18 (uIL-18), released predominantly from proximal tubular cells, has been more extensively validated than plasma IL-18 for detecting renal inflammation. Measurement of uIL-18 offers a window into tubular injury and has been studied extensively as a biomarker for AKI. In patients with ADHF, elevated uIL-18 has been independently associated with a higher risk of AKI development [109]. One multicenter study found that patients with higher uIL-18 levels had a 3.6-fold increased risk of AKI compared to those with lower levels, after adjusting for clinical confounders [110]. Despite these associations, IL-18 lacks organ specificity, as levels may also rise in systemic inflammatory states, infections, and autoimmune disease. Its predictive performance improves when interpreted alongside complementary markers of tubular injury (e.g., NGAL, KIM-1), supporting its use within a biomarker panel rather than as a stand-alone test [109].

#### 4.3.2. Soluble Suppression of Tumorigenicity-2 (sST2)

Soluble ST2 (sST2) is a circulating isoform of the ST2 glycoprotein, a member of the interleukin-1 receptor family. It functions as a decoy receptor for interleukin-33 (IL-33), a cytokine released by damaged or stressed cells that plays a protective role in the heart by reducing inflammation and fibrosis. By binding IL-33 and preventing its interaction with membrane-bound ST2 receptors, sST2 neutralizes its cardioprotective effects and contributes to adverse cardiac remodeling. As such, elevated sST2 levels are considered a marker of myocardial stress, inflammation, and fibrosis [111].

In HF, sST2 has been extensively validated as a prognostic biomarker [112,113]. Unlike natriuretic peptides, sST2 is not significantly affected by age, renal function, or body mass index, making it particularly attractive for risk stratification. Elevated sST2 levels at hospital admission have been associated with increased mortality and rehospitalization in both acute and chronic HF settings [114,115]. Studies suggest that serial monitoring of sST2 may provide additional prognostic information, as a reduction in sST2 after treatment is linked to improved outcomes, independent of changes in NT-proBNP [113]. In clinical practice, sST2 concentrations are most often measured using the Presage^®^ ST2 assay, which is FDA-cleared and widely used to guide decision-making. Levels <35 ng/mL are associated with low likelihood of acute HF, while values >70 ng/mL reflect significant neurohormonal activation and fibrosis, supporting the need for hospitalization and intensified therapy. In addition, a relative reduction of >30% during hospitalization has been proposed as a treatment response signal. These thresholds are intended as prognostic and triage guides rather than diagnostic absolutes (Table 2) [112,116,117].

In patients with CRS, sST2 also holds promise. It has been associated with both cardiovascular events and the development of chronic kidney disease [15]. In individuals with renal dysfunction, elevated sST2 levels predict progression of HF and adverse outcomes, suggesting that it may serve as an integrative biomarker across the heart–kidney axis [118,119]. Moreover, in cardiac intensive care unit settings, combining sST2 with other markers such as NT-proBNP has improved the prediction of AKI and in-hospital events [111].

In summary, sST2 is a robust, dynamic biomarker that reflects myocardial fibrosis and inflammation and offers independent prognostic value in HF. Its utility extends to patients with renal dysfunction and CRS, where it may aid in risk stratification and guide therapeutic decisions [111,113].

#### 4.3.3. Galectin-3 (Gal-3)

Galectin-3 (Gal-3) is a 30-kDa β-galactoside-binding lectin encoded by the LGALS3 gene and secreted mainly by activated macrophages. It plays key roles in inflammation, immune regulation, cell proliferation, and fibrosis [111]. Unlike many cardiac biomarkers, Gal-3 is not heart-specific and is broadly expressed across epithelial and immune cells, including in the kidney and vascular endothelium. Its ability to bind carbohydrate structures allows it to mediate both cell–cell and cell–matrix interactions, contributing to tissue remodeling and fibrotic responses [120].

In HF, Gal-3 has gained attention as a biomarker of myocardial fibrosis and adverse remodeling. Elevated Gal-3 levels have been associated with higher morbidity and mortality across a wide spectrum of cardiovascular conditions, including acute and chronic HF, myocardial infarction, and congenital heart disease [111,113]. Several studies have reported that higher Gal-3 levels correlate with HF severity and predict adverse outcomes independently of natriuretic peptides [111,113]. Unlike NT-proBNP, Gal-3 levels are less influenced by hemodynamic stress and may better reflect structural and inflammatory changes. However, emerging data suggest that its prognostic value may depend on renal function. In one study, Gal-3 levels lost their predictive utility in patients with preserved kidney function, while remaining informative in those with reduced eGFR. The proposed mortality risk thresholds were ~20 ng/mL for patients with eGFR ≥ 60 mL/min/1.73 m^2^ and ~30 ng/mL for those with eGFR < 60 mL/min/1.73 m^2^, underscoring the need to interpret Gal-3 in the context of renal status (Table 2) [121,122].

Gal-3 levels are also elevated in patients with both acute and chronic kidney disease (CKD), often increasing with declining renal function [113,121]. It is believed to contribute to tubulointerstitial fibrosis and inflammation, and its expression has been linked to immune cell infiltration and proteinuria in glomerular diseases. In translational studies, Gal-3 has shown strong associations with AKI severity, proteinuria burden, and progression of CKD, including in patients with diabetes, cardiovascular disease, and autoimmune nephropathies. Both serum and urinary Gal-3 concentrations have demonstrated predictive value for AKI in intensive care, cardiac surgery, and sepsis settings, with area under the curve (AUC) values exceeding 0.85 in some cohorts [120].

In HF patients, Gal-3 appears to have both diagnostic and prognostic utility, but its predictive accuracy may decline in the presence of renal dysfunction [121]. This overlap reflects its dual involvement in both cardiac and renal fibrosis and complicates its interpretation as a stand-alone marker. However, when used alongside other biomarkers such as NT-proBNP or NGAL, Gal-3 can enhance risk stratification, particularly in identifying patients at risk for long-term cardiorenal deterioration. By contrast, its short-term utility for guiding decongestion is limited compared with markers such as sST2 or bio-ADM [121,123].

Overall, Galectin-3 reflects underlying fibrotic and inflammatory processes common to both the heart and kidney. While it is not specific to one organ system, its broad involvement in cardiorenal pathophysiology makes it a valuable biomarker in identifying high-risk patients and potentially guiding antifibrotic or anti-inflammatory therapeutic strategies. Further studies are needed to validate its role in treatment monitoring and to clarify its utility across varying stages of cardiorenal disease [113,120].

#### 4.3.4. Fibroblast Growth Factor-23 (FGF-23) and Klotho Axis

FGF-23 is a 32-kDa hormone primarily secreted by osteocytes and osteoblasts in the bone. Its primary physiological role is to maintain phosphate homeostasis and regulate vitamin D metabolism by acting on the kidneys. FGF-23 requires the presence of the co-receptor α-klotho to activate fibroblast growth factor receptors (FGFRs), most abundantly expressed in the renal tubules. In this context, FGF-23 reduces phosphate reabsorption in the proximal tubules and suppresses the production of 1,25-dihydroxyvitamin D. However, in chronic kidney disease (CKD), FGF-23 levels rise early, often before overt changes in phosphate or parathyroid hormone (PTH) levels, representing one of the first signs of disrupted mineral metabolism and bone mineral disease [124]. In advanced CKD, FGF-23 levels can increase up to 1000-fold and become maladaptive, contributing to systemic effects beyond mineral metabolism, particularly on the cardiovascular system [124,125]. Emerging data suggest that an imbalance between FGF-23 and Klotho may activate Wnt/β-catenin signaling, a pathway involved in cardiac hypertrophy, fibrosis, and endothelial dysfunction. These effects are thought to be mediated through Klotho-independent activation of FGFR4 in the myocardium, leading to cardiomyocyte hypertrophy, calcium dysregulation, and adverse remodeling. Klotho deficiency, common in CKD, appears to amplify these effects. Cross-talk with the renin–angiotensin system may further contribute to volume overload and blood pressure dysregulation [125,126].

Several studies have linked elevated FGF-23 levels to both HF with preserved ejection fraction (HFpEF) and HF with reduced ejection fraction (HFrEF), especially in individuals with CKD. In the CRIC cohort, FGF-23 independently predicted incident HF across all ejection fraction (EF) subtypes, reinforcing its value as a cardiorenal connector. Mechanistically, FGF-23 may promote adverse remodeling through LVH and inflammation, and may indirectly impair cardiac function by reducing Klotho expression [127].

From a diagnostic perspective, FGF-23 holds promise as a biomarker for early CKD-related cardiovascular risk, but its implementation in clinical practice is limited by assay variability (e.g., intact vs. C-terminal FGF-23 assays), lack of standardized cutoffs, and the complexity of its dual endocrine and paracrine actions. Furthermore, while observational data are strong, causal inference remains uncertain, as most evidence is derived from observational cohorts or post hoc analyses rather than randomized controlled trials [124,125,128,129]. Indirect evidence of benefit comes from studies in hemodialysis patients treated with phosphate binders and calcimimetics. In the EVOLVE post hoc analysis, a ≥30% reduction in FGF-23 with cinacalcet was associated with lower cardiovascular event rates and mortality [130]. Similarly, in randomized trials with etelcalcetide, potent FGF-23 suppression (~75%) was linked to reduced progression of left ventricular hypertrophy compared to alphacalcidol, despite equivalent parathyroid hormone control [131].

Additionally, fibroblast growth factor-21 (FGF-21), another member of the same family, has emerged as a stress-induced hormone with potential relevance in CRS. While it may exert counterregulatory effects by reducing oxidative stress and fibrosis, elevated FGF-21 levels likely reflect underlying metabolic stress and disease severity, serving more as a biomarker of poor prognosis rather than a direct mediator, based on current evidence [132,133,134].

In summary, FGF-23 is a pivotal marker and possible mediator in the interplay between kidney dysfunction and heart failure. Its early elevation in CKD and its strong association with adverse cardiovascular outcomes highlight its potential utility in risk stratification and possibly future therapeutic strategies in cardiorenal syndrome. Though assay heterogeneity and uncertain causality currently limit its clinical application [125,126,135].

#### 4.3.5. Growth Differentiation Factor-15 (GDF-15)

GDF-15 is a stress-responsive cytokine belonging to the transforming growth factor-beta (TGF-β) superfamily. Under physiological conditions, it is weakly expressed across tissues, with higher expression in the placenta and low baseline levels in circulation. However, its expression rises markedly in response to cellular stress, tissue injury, inflammation, and oxidative stress [136,137]. Although initially characterized for its anti-inflammatory effects on macrophages, GDF-15 is now recognized as a pleiotropic molecule involved in diverse pathological states, including cardiovascular diseases, kidney disease, cancer, and metabolic disorders. Importantly, circulating GDF-15 levels are strongly influenced by non-disease factors such as age, smoking, cancer, and exposure to medications including metformin and chemotherapy. These confounders must be considered when interpreting elevated values in clinical practice [136,138].

In kidney pathophysiology, both plasma and urinary GDF-15 levels increase in acute and chronic kidney disease, reflecting ongoing inflammation, oxidative stress, and tissue injury [136]. Experimental studies suggest that GDF-15 may exert protective renal effects by limiting fibrosis, reducing oxidative stress, and preserving tubular integrity, partly through modulation of the PI3K/Akt and NF-κB pathways [136,139]. However, in clinical settings, elevated GDF-15 levels typically signal worse outcomes. Higher serum GDF-15 concentrations have been independently associated with CKD progression, the need for renal replacement therapy, and adverse cardiovascular events [140,141,142,143,144].

In heart failure, GDF-15 is considered a robust prognostic marker. Elevated levels correlate with worse outcomes across both preserved and reduced ejection fraction phenotypes and are particularly predictive in patients with coexisting CKD [137,141,142,145]. GDF-15 levels have also been linked to right ventricular dysfunction, congestion, and cachexia in advanced heart failure, reflecting its association with systemic metabolic stress [143]. Notably, serial measurements of GDF-15, especially when combined with natriuretic peptides like NT-proBNP, improve risk stratification in acute heart failure, outperforming single time-point assessments. While dedicated studies on GDF-15 in CRS are limited, its dual association with cardiac and renal dysfunction, as well as inflammation and fibrosis, highlights its potential as a unifying biomarker in CRS and warrants further investigation [137,142].

Despite its predictive power, the interpretation of GDF-15 remains complex. As a systemic stress marker, it lacks organ specificity, and its exact role—whether adaptive or maladaptive—within the cardiovascular and renal systems is still under investigation [136]. Elevated GDF-15 may reflect both the severity of the underlying disease and a compensatory response to injury. Additionally, its levels increase with age, smoking, cancer, and certain treatments like metformin or chemotherapy, which can confound clinical interpretation [136,138].

In summary, GDF-15 is a promising biomarker in cardiorenal syndrome, capturing the systemic stress and inflammatory burden that accompany disease progression. While it enhances prognostic evaluation, particularly when combined with other biomarkers, its non-specific nature and susceptibility to confounding factors currently limit its use as a stand-alone tool, reinforcing its value as part of a multi-marker strategy [138].

### 4.4. Markers of Congestion

#### 4.4.1. Carbohydrate Antigen 125 (CA125)

Carbohydrate antigen 125 (CA125), also known as MUC16, is a high-molecular-weight transmembrane glycoprotein originally recognized as a tumor marker in ovarian and colorectal cancer. Beyond oncology, CA125 is physiologically expressed by mesothelial cells lining the pericardium, pleura, and peritoneum, where it contributes to epithelial lubrication and defense against mechanical stress. In heart failure (HF), particularly in the context of right-sided dysfunction and systemic congestion, CA125 levels rise in response to mechanical stretch of mesothelial surfaces and inflammatory stimuli. Venous congestion leads to serosal effusions, bowel wall edema, and endothelial activation, which together with the release of pro-inflammatory cytokines (IL-6, IL-10, etc.) stimulate CA125 production [146,147].

Unlike natriuretic peptides, CA125 levels are less influenced by left ventricular ejection fraction (LVEF), renal function, age, or obesity, making it a distinct biomarker of tissue congestion rather than intravascular volume status [146,148]. In acute HF, elevated CA125 (>35 U/mL) correlates with congestion severity, right ventricular dysfunction, pleural effusions, and peripheral edema (Table 2). This marker has demonstrated prognostic utility, predicting mortality and rehospitalization independently of natriuretic peptides, particularly in patients with CRS or renal dysfunction where NT-proBNP loses specificity. Importantly, CA125 demonstrates on-treatment kinetics. Levels typically fall with effective decongestive therapy, and a lack of decline—or a rise post-discharge—signals persistent or recurrent congestion. In such cases, clinical reassessment, diuretic uptitration, or closer follow-up should be considered [146,148,149,150,151].

Serial measurement of CA125 provides insight into decongestion status and residual congestion after hospital discharge. Clinical studies, including the CHANCE-HF trial, have shown that CA125-guided therapy—primarily in adjusting diuretic strategies—reduces HF readmissions [152]. Persistently elevated or rising CA125 levels after discharge are associated with worse outcomes. In chronic HF and HF with preserved ejection fraction (HFpEF), higher CA125 correlates with atrial enlargement, impaired renal function, and reduced exercise capacity [148,149]. However, CA125 is not heart-specific and can be elevated in non-cardiac conditions such as malignancies, liver cirrhosis, peritoneal inflammation, and infections. Therefore, interpretation requires a comprehensive clinical assessment. Moreover, while it effectively reflects serosal and tissue congestion, CA125 is less sensitive to isolated intravascular congestion [146,149].

In clinical practice, CA125 serves as a complementary tool to natriuretic peptides and imaging, especially useful in elderly patients, those with renal dysfunction, or where right-sided HF predominates. A baseline and follow-up measurement post-decongestion can help stratify risk, optimize therapy, and potentially improve outcomes. While further research is needed to standardize its use, current evidence supports the clinical value of CA125 in assessing congestion and guiding management in HF and CRS [146,153].

#### 4.4.2. Bioactive Adrenomedullin (Bio-ADM) and Mid-Regional Pro-Adrenomedullin (MR-ProADM)

Adrenomedullin (ADM) is a vasodilatory peptide secreted predominantly by vascular endothelial cells in response to volume overload, inflammation, and endothelial stress. Its biologically active form, bio-ADM, directly reflects vascular integrity and endothelial stress, but it is technically challenging to measure in routine practice. The stable, inactive fragment midregional pro-adrenomedullin (MR-proADM) is more widely used in clinical research due to its stability, yet both forms reflect vascular and tissue congestion in HF [154,155].

Elevated bio-ADM levels have been shown to correlate with clinical and hemodynamic markers of congestion, such as peripheral edema, orthopnea, and elevated right atrial pressures [156,157]. Bio-ADM also correlates with pulmonary capillary wedge pressure and right atrial mean pressure. A cut-off of 34 pg/mL has been proposed in research cohorts for identifying significant congestion, but it is not guideline-endorsed (Table 2) [158]. Similarly, MR-proADM is associated with pulmonary artery pressures and inversely with pulmonary artery compliance, particularly in HFpEF [158]. MR-proADM has demonstrated superior accuracy compared to natriuretic peptides in predicting 90-day and 1-year mortality in HF cohorts [155]. Importantly, bio-ADM levels remain elevated throughout hospitalization for acute HF and are modestly predictive of mortality and rehospitalization. Combining bio-ADM with NT-proBNP may enhance prognostic precision compared to either biomarker alone [15]. The STRONG-HF trial further emphasized that reductions in bio-ADM tracked with clinical decongestion over time [156]. Beyond its biomarker role, bio-ADM is under investigation as a therapeutic target. Adrecizumab, a non-neutralizing monoclonal antibody, binds circulating bio-ADM, shifting it from the interstitium to the vasculature, potentially enhancing vascular integrity [154]. Ongoing trials are assessing its role in conditions marked by severe interstitial congestion, such as acute HF and sepsis.

In clinical practice, while bio-ADM and MR-proADM are not yet part of standard care, MR-proADM is more feasible for clinical adoption due to its stability and availability, while bio-ADM offers stronger mechanistic insight but limited accessibility. Both show promise as adjuncts for evaluating residual congestion, particularly post-discharge, where they may guide diuretic strategies and risk stratification in CRS [155].

#### 4.4.3. Copeptin (CPP)

Copeptin is the C-terminal part of the arginine vasopressin (AVP) precursor and is released in equimolar amounts with AVP, making it a reliable surrogate marker of vasopressin activity. AVP plays a crucial role in fluid homeostasis, urine osmolarity, vasoconstriction, and stress responses, but its direct measurement is limited by its instability and short half-life. In contrast, copeptin is highly stable, easily measurable in plasma or serum, and unaffected by factors such as platelet binding or circadian rhythm, rendering it clinically practical [159,160].

The release of copeptin is triggered by osmotic, hemodynamic, and stress stimuli. As such, copeptin reflects systemic stress, neurohormonal activation, and volume status—pathophysiological hallmarks of HF, kidney disease, and the broader cardiorenal-metabolic spectrum. In healthy individuals, copeptin inversely correlates with glomerular filtration rate (GFR), and elevated levels are consistently observed in CKD, cardiovascular diseases (CVD), and metabolic disorders such as diabetes and obesity [159,161].

In HF, copeptin levels increase with disease severity, correlate with NYHA class, and have been associated with worse cardiac function, particularly in HFrEF. Studies have shown that copeptin predicts mortality and rehospitalization in acute and chronic HF, often outperforming traditional markers like NPs in short-term prognostic settings particularly among patients presenting with acute dyspnea. Copeptin also shows potential as a prognostic marker in patients with CRS, where it may reflect the compounded stress and fluid dysregulation between heart and kidney dysfunction [160,162].

Nevertheless, the clinical utility of copeptin remains somewhat limited by several factors. While it consistently correlates with adverse outcomes, its incremental prognostic value beyond established markers such as NT-proBNP is modest and inconsistent across studies. Studies have yielded inconsistent results regarding its ability to guide therapy, such as beta-blocker titration or vasopressin antagonism. Moreover, copeptin levels can rise in various non-cardiac conditions (e.g., sepsis, stroke, AKI), which may confound its interpretation in HF populations [159,160].

In summary, copeptin is a stable, easily measurable biomarker reflecting AVP-driven neurohormonal activation and systemic stress, with consistent associations with HF severity and prognosis. Its role is most promising in risk stratification and research-based multi-marker panels, but its integration into routine clinical practice is constrained by modest add-on value, limited specificity and the absence of therapeutic cut-offs [159,160].

**Table 2:** Proposed cut-off values for selected biomarkers and their suggested clinical applications. This table presents biomarker thresholds derived from key clinical studies, highlighting their potential utility in guiding diagnosis, prognosis, and treatment decisions in heart failure and cardiorenal syndrome. While these values are not yet universally validated or standardized, they offer a practical framework for interpretation in appropriate clinical contexts. Bio-ADM: Bioactive adrenomedullin; CA125: Carbohydrate antigen 125; eGFR: Estimated glomerular filtration rate; HF: Heart failure; IGFBP7: Insulin-like growth factor binding protein 7; sST2: Soluble Suppression of Tumorigenicity-2; TIMP-2: Tissue inhibitor of metalloproteinases-2.

Biomarker	Proposed Cut-Off	Clinical Utility	Study
Bio-ADM	>34 pg/mL	Higher risk of residual congestion following discharge	ter Maaten et al., 2019 [157]
CA125	>35 U/mL	Prognostic value in acute HF Congestion severity	Núñez et al., (CHANCE-HF) [152]
Galectin-3	19.7 ng/mL (eGFR ≥ 60); 31.5 ng/mL (eGFR < 60)	Mortality risk stratification based on kidney function	Caravaca Perez P et al. [121]
TIMP-2/IGFBP7 (NephroCheck^®^)	>2—High AKI risk >0.3—≤2: Moderate AKI risk ≤0.3—Low AKI risk	Early AKI risk stratification and management strategies	Guzzi et al. [101]
sST2	<35 ng/mL	Low likelihood of acute HF	Januzzi et al., 2015 [117] Tang et al., 2016 [112]
	>70 ng/mL	Significant neurohormonal activation/ Fibrosis	
	>30% reduction during hospitalization	Treatment goal for acute HF	

## 5. Micro-RNAs as Emerging Biomarkers and Therapeutic Targets in Cardiorenal Syndrome

MicroRNAs (miRNAs) are small, non-coding RNAs involved in gene regulation and have emerged as promising biomarkers and therapeutic targets in HF, kidney disease, and CRS. Their tissue specificity, stability in circulation, and close association with key pathophysiological processes—such as hypertrophy, fibrosis, inflammation, and metabolic dysfunction—highlight their clinical potential [163,164].

Among them, miR-21 is the most studied, with strong evidence linking it to cardiac and renal fibrosis through pathways like TGF-β and extracellular signal-regulated kinase (ERK)- mitogen-activated protein kinase (MAPK). Its inhibition via antisense oligonucleotides (ASOs) has shown favorable effects in preclinical models of HF, CKD, and CRS. Similarly, other miRNAs, including miR-132 and miR-92a, have been implicated in myocardial remodeling and vascular repair. Circulating miRNAs are detectable in plasma and urine, offering non-invasive diagnostic potential. Distinct miRNA profiles have been associated with HFpEF, myocardial ischemia, and inflammatory cardiomyopathy, suggesting their role in risk stratification and precision medicine [164,165].

Therapeutically, miRNA mimics and inhibitors are under investigation, with clinical trials targeting miR-21 (e.g., in renal fibrosis) and miR-132 (in HF). However, challenges such as targeted delivery, off-target effects, and long-term safety need further research before widespread clinical application. In conclusion, miRNAs represent a promising approach in CRS, offering both diagnostic and therapeutic potential, though more research is needed before they can be used in clinical practice [164,166].

## 6. From Bench to Bedside: Clinical Application of Biomarkers in Cardiorenal Syndrome Management

The clinical utility of biomarkers in cardiorenal syndrome (CRS) lies not in their isolated measurement, but in their integrated interpretation within specific clinical contexts. This section moves beyond individual biomarker descriptions and provides a scenario-based framework for application, highlighting comparative strengths and guiding real-world decision-making [15,51].

### 6.1. Navigating Key Clinical Scenarios

#### Scenario 1: The Inpatient with “Worsening Renal Function”—The Decongestion Dilemma

A central challenge in managing hospitalized patients with CRS is differentiating true WRF—indicating structural kidney injury—from functional, hemodynamically mediated rises in serum creatinine during decongestive therapy. This distinction is especially important in critically ill or advanced heart failure patients, in whom inappropriate treatment withdrawal may compromise outcomes, while delayed recognition of true injury may allow irreversible damage to occur [17,21,22].

Upon presentation to the emergency department or admission unit, early biomarker assessment can help establish a risk baseline. Serum Cr and cystatin C should be interpreted together, particularly in patients with advanced HF. Cystatin C has been shown to be a more accurate estimator of GFR in frail or sarcopenic individuals, where creatinine-based estimates may be misleading. Moreover, a significant discrepancy between creatinine- and cystatin C–derived eGFR may serve as an early indicator of worse prognosis, reflecting heightened neurohormonal activation and impaired renal reserve. These patients typically require careful decongestion with close pharmacological monitoring, as they are more prone to intolerance or side effects of therapy [31,32,34,167].

At this early stage, measurement of renal tubular injury biomarkers—such as plasma or urinary NGAL, urinary KIM-1, and uL-FABP—is advised. Elevated baseline levels suggest ongoing subclinical tubular damage, potentially due to venous congestion, inflammation, or ischemia. Identifying these high-risk patients allows clinicians to initiate more intensive decongestive strategies and to consider earlier initiation of guideline-directed medical therapy (GDMT), aiming to improve outcomes through more timely volume offloading and neurohormonal modulation [20,64,90,91,168].

Once decongestion is initiated, fluctuations in serum creatinine are frequently observed. The interpretation of these fluctuations must be guided by repeated clinical and biomarker assessments. Tubular biomarkers are particularly helpful in this context. A stable or only mildly fluctuating NGAL, KIM-1, or uL-FABP trajectory typically reflects functional hemodynamic changes and supports the continued use of diuretics. In contrast, a marked rise in any of these markers may have different implications depending on timing. In the early phase, it often reflects persistent congestion, indicating the need for more aggressive decongestion and hemodynamic reassessment. In later stages, it is more likely due to overdiuresis, drug-induced nephrotoxicity, or true WRF from hypoperfusion, requiring therapy adjustment, withdrawal of nephrotoxins, and in select cases, consideration of renal replacement therapy [17,81,168,169,170].

Albuminuria represents another important marker with both diagnostic and prognostic significance in this setting. As mentioned before, in HF patients, albuminuria may result from multiple mechanisms, including glomerular damage, impaired tubular reabsorption, and most notably, venous congestion and systemic endothelial dysfunction. Importantly, albuminuria levels tend to improve following decongestive therapy, which can serve as a supportive marker of treatment efficacy. For consistency, UACR is best measured from morning spot urine samples collected at a consistent time after diuretic administration [52,57,58,171].

An emerging tool for early AKI risk prediction is the NephroCheck^®^ test, which combines TIMP-2 and IGFBP-7. These markers reflect early cellular stress and G1 cell-cycle arrest in renal tubular cells—changes that precede overt tubular damage. The test result, expressed as [TIMP-2] × [IGFBP-7] in (ng/mL)^2^/1000, is interpreted along a validated risk continuum. A value below 0.3 indicates a low risk of developing moderate-to-severe AKI (KDIGO stage 2–3) within the following 12–24 h. Values above 2.0 signify a high risk of imminent AKI and should prompt urgent implementation of nephroprotective strategies (hemodynamic optimization, minimization or avoidance of nephrotoxic agents, etc.). While NephroCheck is primarily validated in high-risk populations—such as ICU patients, those undergoing major cardiac surgery, or individuals with septic shock—its clinical application in advanced HF is increasingly recognized. In this context, elevated NephroCheck values may indicate the need for more aggressive decongestion and timely GDMT initiation [101,102,105].

**Figure 1:**
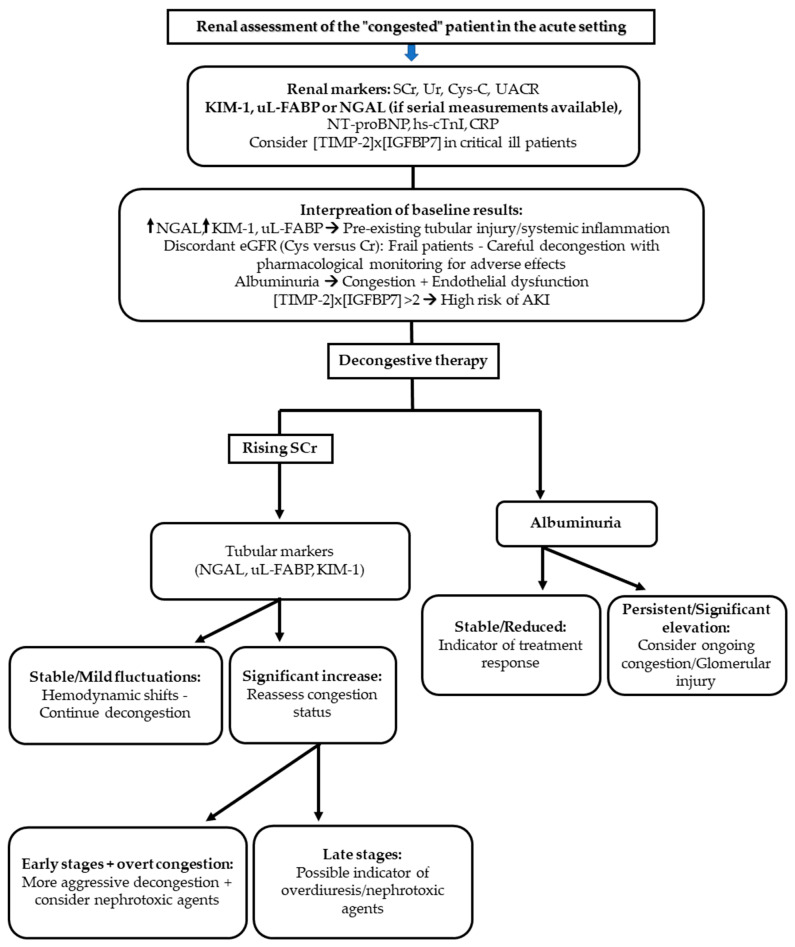
Algorithm for renal assessment of the “congested” patient in the acute setting. Biomarkers complement conventional renal indices to guide diagnosis and management. Initial evaluation includes serum creatinine (SCr), urea (Ur), cystatin C (Cys-C), and urinary albumin-to-creatinine ratio (UACR), with tubular injury markers (NGAL, KIM-1, uL-FABP) and [TIMP-2] × [IGFBP7] considered in selected patients. Interpretation of baseline results enables recognition of pre-existing tubular injury, endothelial dysfunction, and risk of acute kidney injury (AKI). During decongestive therapy, trends in SCr, tubular markers, and albuminuria help differentiate hemodynamic shifts from true injury, distinguish early versus late stages of renal dysfunction, and tailor the intensity of therapy while minimizing nephrotoxic risk. AKI: acute kidney injury; CRP: C-reactive protein; Cys-C: cystatin C; hs-cTnI: high-sensitivity cardiac troponin I; KIM-1: kidney injury molecule-1; NGAL: neutrophil gelatinase-associated lipocalin; NT-proBNP: N-terminal pro-B-type natriuretic peptide; SCr: serum creatinine; TIMP-2 × IGFBP7: tissue inhibitor of metalloproteinases-2 × insulin-like growth factor binding protein-7; UACR: urinary albumin-to-creatinine ratio; Ur: urea; uL-FABP: urinary liver-type fatty acid–binding protein.

In summary, a patient-centered approach that integrates traditional renal markers with tubular injury biomarkers (NGAL, KIM-1, uL-FABP), filtration markers (cystatin C), albuminuria, and early stress markers (NephroCheck) throughout the course of hospitalization can improve diagnostic accuracy, allow safer titration of therapies, and enhance clinical decision-making in patients with CRS and suspected WRF [64,91,99,101,168] (Table 3, Figure 1).

### 6.2. Scenario 2: The Acutely Dyspneic Patient: Diagnosing and Stratifying Congestion

In the emergency setting, a patient presenting with acute dyspnea requires rapid differentiation between cardiac and non-cardiac causes. Clinical examination remains the first step, with key findings such as orthopnea, rales, jugular venous distention, and peripheral edema pointing toward heart failure. These signs can be quickly corroborated by point-of-care ultrasound, using IVC size and collapsibility, lung B-lines, or renal venous Doppler to provide an immediate picture of intravascular and extravascular fluid overload [169,172].

At this stage, biomarkers refine the diagnostic certainty and stratify congestion. Soluble ST2 (sST2) is particularly useful to confirm that dyspnea is cardiac in origin. Low levels (<35 ng/mL) make acute HF unlikely, while values >70 ng/mL indicate marked myocardial stress and fibrosis, supporting the need for hospital admission and intensive therapy. Beyond diagnosis, a reduction of >30% in sST2 during hospitalization has been associated with improved outcomes, offering a practical therapeutic target [117].

Once acute HF is established, the focus shifts to characterizing the congestion status. Carbohydrate Antigen 125 (CA125) is a robust marker of tissue-level and serosal fluid overload, especially in right-sided HF or when natriuretic peptides are less informative due to renal dysfunction or preserved ejection fraction. Elevated levels (>35 U/mL) identify high-risk patients and, importantly, serial measurements track decongestion and recovery, guiding both inpatient therapy and follow-up intensity [146,152].

To further define systemic and vascular congestion, bio-ADM and its stable fragment MR-proADM provide insight into endothelial dysfunction and hemodynamic burden. High levels (>34 pg/mL) correlate with elevated filling pressures, persistent fluid overload, and increased mortality risk, particularly in HF with preserved EF. In cases where natriuretic peptides are inconclusive, bio-ADM adds complementary information, and when combined with NT-proBNP it strengthens both diagnostic and prognostic confidence [155,156,157].

**Figure 2:**
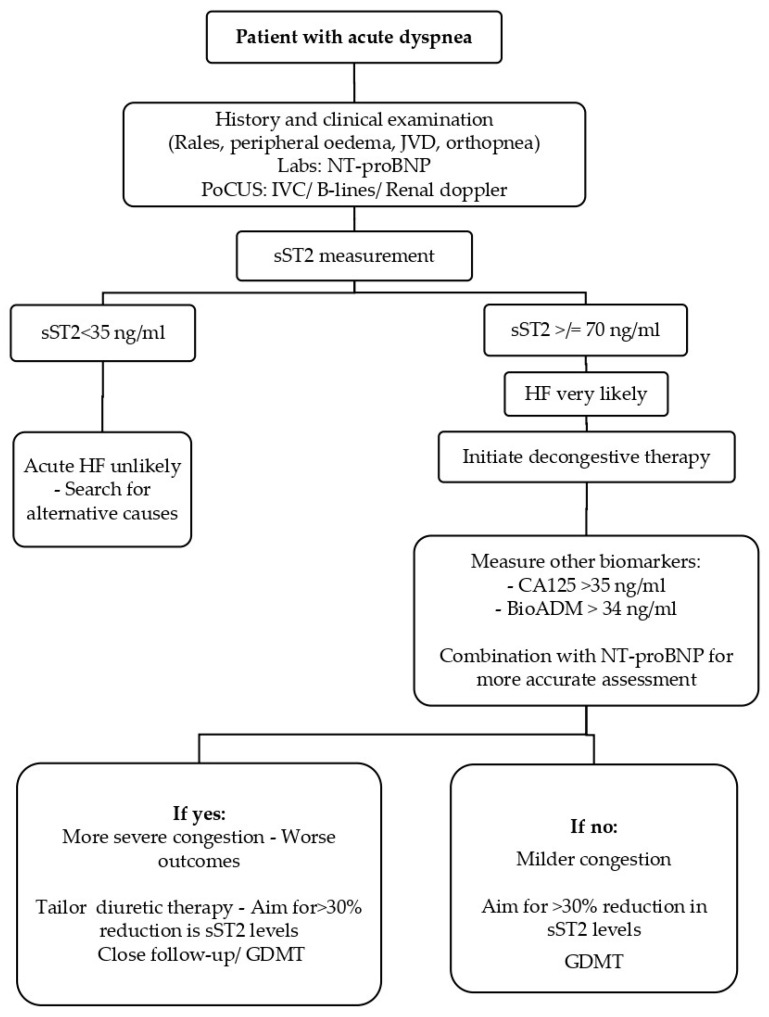
Biomarker-guided algorithm for the patient with acute dyspnea. Clinical evaluation—including history, physical examination (rales, peripheral edema, jugular venous distension, orthopnea), laboratory testing (NT-proBNP), and point-of-care ultrasound (IVC, B-lines, renal Doppler)—is the first step in assessing a patient presenting with acute dyspnea. Measurement of soluble ST2 (sST2) refines the diagnostic likelihood of acute heart failure (HF): values < 35 ng/mL make HF unlikely, whereas levels ≥ 70 ng/mL indicate high probability and justify initiation of decongestive therapy. Additional biomarkers such as CA125 (>35 U/mL) and bioactive adrenomedullin (BioADM > 34 ng/mL), particularly in combination with NT-proBNP, help stratify congestion severity and prognosis. Tailoring therapy toward >30% reduction in sST2 levels, alongside guideline-directed medical therapy (GDMT), improves risk stratification and guides follow-up intensity. BioADM: bioactive adrenomedullin; CA125: carbohydrate antigen 125; GDMT: guideline-directed medical therapy; HF: heart failure; IVC: inferior vena cava; JVD: jugular venous distension; NT-proBNP: N-terminal pro-B-type natriuretic peptide; PoCUS: point-of-care ultrasound; sST2: soluble suppression of tumorigenicity-2.

In summary, the acutely dyspneic patient benefits from a layered, clinically integrated approach. Bedside examination and ultrasound guide the initial impression, while biomarkers provide precision: sST2 confirms the likelihood of acute HF, CA125 quantifies tissue and serosal congestion, and bio-ADM highlights vascular dysfunction. Used together, they help clinicians not only establish the diagnosis but also individualize therapy and monitoring, ensuring that treatment intensity matches the true burden of congestion (Figure 2, Table 3) [152,156].

### 6.3. Scenario 3: The Chronic Patient—Long Term Risk Stratification and Management

In stable outpatients, biomarkers help clinicians move beyond short-term decongestion and toward long-term risk assessment and tailored management. For prognostic enrichment, biomarkers reflecting inflammation such as IL-18 and GDF-15 highlight systemic processes that may drive progression of both heart and kidney failure. Their elevation should prompt not only optimization of GDMT for HF but also strict control of comorbidities, autoimmune activity, and lifestyle factors (salt, alcohol, smoking, and physical inactivity) [106,108,136,142].

Markers of fibrosis such as Galectin-3 and FGF-23 provide additional layers of information. Galectin-3 reflects cardiac and renal fibrosis, while FGF-23 has been associated with adverse ventricular remodeling and neurohormonal activation. Serial measurement of FGF-23, in particular, may offer insight into response to chronic HF therapies or device-based interventions such as cardiac resynchronization therapy [121,129,135].

Finally, proenkephalin (PENK) is emerging as a marker of neurohormonal activation and sympathetic drive. Elevated levels may support prioritizing therapies targeting the renin–angiotensin–aldosterone system, including ARNIs, ACE inhibitors/ARBs, and MRAs [47,50].

In summary, in the chronic HF outpatient, biomarkers complement clinical assessment by refining CKD classification, identifying inflammatory and fibrotic risk, and signaling neurohormonal activation. Together, they allow for a more individualized approach to long-term therapy and follow-up, ensuring that treatment intensity and monitoring are aligned with each patient’s evolving biological risk profile (Table 3).

### 6.4. Practical Considerations: Cost, Turnaround Time and Availability

The use of novel biomarkers in cardiorenal syndrome faces not only interpretative challenges but also practical and economic barriers. Formal cost-effectiveness analyses are almost entirely lacking, leaving a major evidence gap that hinders reimbursement and routine use [173,174].

#### 6.4.1. Availability

CA-125 is universally accessible, and Cystatin C is increasingly routine. NephroCheck^®^ and sST2 are FDA-cleared but remain limited to specialized centers. Most other markers (NGAL, KIM-1, L-FABP, bio-ADM, PENK) are largely confined to research with limited clinical utility [15,174].

#### 6.4.2. Turnaround Time

NephroCheck^®^ is rapid (<1 h), enabling point-of-care decisions [175,176]. Cystatin C and sST2 generally return results within a day, while research-based assays often take >24 h and are unsuitable for acute management [33,64,111].

#### 6.4.3. Cost

Standard assays (CA-125, Cystatin C) are comparatively less expensive, whereas patented tests such as NephroCheck^®^ and sST2 are considerably more costly, and their use should be justified by a demonstrable impact on patient management and outcomes (Table 4) [174,176].

In summary, novel biomarkers currently fit best in tertiary care or clinical trial settings. Limited availability, variable turnaround time, and higher costs demand careful selection of context, while future research must address not only clinical validity but also economic value to enable broader adoption [15,174].

## 7. Conclusions and Future Perspectives

Cardiorenal syndrome (CRS) remains a major clinical challenge due to the complex and bidirectional interplay between cardiac and renal dysfunction, which conventional biomarkers like creatinine and natriuretic peptides fail to fully capture. This review has synthesized the evidence for a new generation of biomarkers that illuminate specific pathophysiological pathways—including glomerular filtration, tubular injury, congestion, inflammation, and fibrosis—providing a more nuanced understanding of CRS heterogeneity.

The clinical utility of these markers is maximized not in isolation, but when integrated into a scenario-based framework that addresses common dilemmas: differentiating structural renal injury from hemodynamically mediated creatinine changes during decongestion, unmasking tissue-level congestion in cases of diagnostic uncertainty, and refining long-term prognostic stratification. The combined application of biomarkers such as NGAL, KIM-1, uL-FABP, CA125, and sST2 offers a powerful strategy to move beyond the limitations of traditional tests, enabling more precise and personalized patient management.

However, significant barriers to routine implementation remain. These include the non-specificity of certain markers, a critical lack of robust health-economic data on cost-effectiveness, limited assay availability outside specialized centers, and an absence of standardized, context-specific cut-off values. To overcome these hurdles and translate promise into practice, future efforts must prioritize three key areas: (1) the design of prospective, biomarker-guided randomized controlled trials that test specific management algorithms against standard care; (2) the development and validation of accessible, rapid-turnaround assays suitable for point-of-care decision-making; and (3) the exploration of novel tools, such as microRNAs, for their dual diagnostic and therapeutic potential.

In conclusion, the journey from biomarker discovery to bedside application in CRS is ongoing. By embracing a multi-marker, phenotype-informed approach and addressing the extant practical and evidence-based gaps, the future management of CRS can evolve from reactive to proactive, ultimately improving outcomes for this high-risk population. While this review has focused primarily on biomarkers in acute and chronic cardiorenal syndromes, future research should also extend to renocardial processes, such as left ventricular hypertrophy in CKD and cardiac complications of dialysis, which remain underexplored.

## Figures and Tables

**Table 1:** Key points for the use of Cystatin-C in patients with heart failure [15,17,28,31,34,35].

Clinical Use of Cystatin-C in Cardiorenal Syndrome—When and Why:
**What it offers:** Better surrogate marker of glomerular filtration rate compared to serum creatinine levels—Less dependent on age, nutritional status or muscle mass
**When to use:** Consider cystatin C measurement in the following clinical scenarios: ■ Advanced heart failure or critical illness ■ Sarcopenia, cachexia, frailty, Hepatic cirrhosis ■ When creatinine-based GFR may overestimate renal function
**Important caveats:** ■ Thyroid dysfunction ■ Obesity ■ Inflammatory conditions ■ Corticosteroid use
**Prognostic value:** A large discrepancy between eGFR from the combined equation and the creatinine-only has been associated with worse outcomes and may indicate advanced disease
**Direct GFR measurement:** In clinical situations where highly accurate GFR is essential (e.g., kidney transplantation, clinical trials etc.), direct GFR measurements with exogenous filtration remain the gold standard.

**Table 3:** Summary of selected biomarkers in CRS: clinical utility, diagnostic strengths, limitations, and practical considerations. AKI: Acute kidney injury; bio-ADM: Bioactive adrenomedullin; CA125: Carbohydrate antigen 125; CKD: Chronic kidney disease; Gal-3: Galectin-3; GDF-15: Growth differentiation factor-15; GFR: Glomerular filtration rate; ICU: Intensive Care Unit; IGFBP7: Insulin-like growth factor binding protein 7; IL-18: Interleukin-18; KIM-1: Kidney injury molecule-1; L-FABP: Liver-type Fatty Acid Binding Protein; MR-proADM: Mid-regional pro-adrenomedullin; NT-proBNP: PENK: Proenkephalin; sST2: Soluble Suppression of Tumorigenicity-2, TIMP-2: Tissue inhibitor of metalloproteinases-2.

Clinical Setting	Recommended Biomarkers	Primary Role	Comments
**Acute Kidney Injury (AKI)**	NGAL, KIM-1, uL-FABP, [TIMP-2] × [IGFBP7]	Early detection of tubular injury	Best used in acute HF, perioperative or ICU settings
**Chronic Kidney disease (CKD)**	Serum Cystatin C, urine β2-Microglobulin, Galectin-3, GDF-15	GFR estimation, fibrosis, prognosis	Cystatin C preferred in frail/sarcopenic patients; Gal-3 useful in proteinuric CKD
**Congestion (Residual/Chronic)**	CA125, bio-ADM, MR-proADM, UACR, sST2	Tissue and vascular congestion	Serial monitoring guides diuretic strategy
**Prognosis**	sST2, GDF-15, Galectin-3, MR-proADM, UACR, Copeptin	Long-term outcomes	Combine with NT-proBNP or imaging for best risk stratification

**Table 4:** Availability, turnaround time, and approximate cost of selected biomarkers relevant to cardiorenal syndrome. CA-125 and Cystatin C are widely available and relatively inexpensive, whereas soluble ST2 (sST2) and NephroCheck^®^ remain more specialized, higher-cost assays with limited accessibility. Turnaround time varies from <1 h (NephroCheck^®^) to >24 h for many research-based assays. The table underscores the resource-dependent nature of biomarker testing, emphasizing that selection must be tailored to the clinical setting and cost justification CA-125: Carbohydrate antigen 125; ELISA: Enzyme-linked immunosorbent assay; IGFBP7: Insulin-like growth factor binding protein-7; POC: Point-of-care; sST2: Soluble suppression of tumorigenicity-2; TIMP-2: Tissue inhibitor of metalloproteinases-2; USD: United States dollars.

Biomarker	Typical Availability	Turnaround Time	Relative Cost
CA-125	Routine in most labs	1–2 business days in many settings	Low
Cystatin C	Increasingly available in hospitals and reference labs	Same day to next day (core lab)	Low-Moderate
sST2 (ELISA-kit research level)	More specialized/major reference labs or for research	Several hours to a day (if batch processing)	Moderate-High
NephroCheck^®^ (TIMP-2 × IGFBP7)	Specialized centers; high cost; less publicly listed price info	<1 h turnaround in many settings (POC or rapid core lab)	High
NGAL	Primarily research-based; limited routine use	Rapid	Moderate- High
KIM-1 uL-FABP	Primarily research-based; limited routine use	Typically batch tested (>24 h); rapid assays in development	High

## Data Availability

No new data were created or analyzed in this study. Data sharing is not applicable to this article.

## Abbreviations

The following abbreviations are used in this manuscript:

ADHF	Acute decompensated heart failure
AF	Atrial Fibrillation
AKI	Acute kidney injury
ASOs	Antisense oligonucleotides
AVP	Arginine vasopressin
bio-ADM	Bioactive adrenomedullin
BNP	B-type natriuretic peptide
CA125	Carbohydrate antigen 125
CAD	Coronary artery disease
CHARM	Candesartan in Heart Failure—Assessment of Reduction in Mortality and Morbidity
CHART-2	Chronic Heart Failure Analysis and Registry in the Tohoku District 2
CKD	Chronic kidney disease
CMIA	Chemiluminescent microparticle immunoassay
CPP	Copeptin
Cr	Creatinine
CRS	Cardiorenal syndrome
CV	Cardiovascular
EF	Ejection fraction
eGFR_Cr_	Creatinine-based estimated glomerular filtration rate
eGFR_Cys_	Cystatin-based estimated glomerular filtration rate
ELISA	Enzyme-linked immunosorbent assay
ERK	Extracellular signal-regulated kinase
ESRD	End-Stage Renal disease
FDA	Food and Drug Administration
FGF-21	Fibroblast Growth Factor-21
FGF-23	Fibroblast Growth Factor-23
FGFRs	Fibroblast Growth Factor receptors
Gal-3	Galectin-3
GBM	Glomerular basement membrane
GDF-15	Growth differentiation factor-15
GDMT	Guideline directed medical therapy
GFR	Glomerular filtration rate
GLP-1 RA	Glucagone-like-peptide-1 receptor agonists
HF	Heart failure
HFpEF	Heart failure with preserved ejection fraction
HFrEF	Heart failure with reduced ejection fraction
Hc-cTnI	High-sensitive cardiac troponin I
ICU	Intensive Care Unit
IFN-γ	Interferon-γ
IGFBP7	Insulin-like growth factor binding protein 7
IL-1	Interleukin-1
IL-18	Interleukin-18
IL-18BP	Interleukin-18 binding protein
IL-33	Interleukin-33
IVC	Inferior vena cava
KCCQ	Kansas City Cardiomyopathy Questionnaire
KIM-1	Kidney Injury Molecule-1
L-FABP	Liver-type Fatty Acid Binding Protein
LVEF	Left ventricular ejection fraction
LVH	Left ventricular hypertrophy
MAPK	Mitogen-activated protein kinase
miRNAs	MicroRNAs
miR-21	MicroRNA-21
MMP-9	Metalloproteinase-9
MR-ProADM	Mid-regional pro-adrenomedullin
MyD88	Myeloid differentiation factor 88
NF-kB	Nuclear factor kappa B
NGAL	Neutrophil gelatinase associated lipocalin
Ns-MRAs	Non-steroidal mineralocorticoid receptors antagonists
NPs	Natriuretic peptides
NT-proBNP	N-terminal pro-B-type natriuretic peptide
PARADIGM-HF	Prospective comparison of ARNI with ACEI to Determine Impact on Global Mortality and morbidity in Heart Failure
PENK	Proenkephalin
PoCUS	Point of care ultrasound
PTH	Parathyroid hormone
RAAS	Renin–Angiotensin–Aldosterone System
SGLT2i	Sodium-glucose cotransporter-2 inhibitors
SNGFR	Single-nephron glomerular filtration rate
sST2	Soluble Suppression of Tumorigenicity-2
ΤAΤ	Turnaround time
TGF-β	Transforming growth factor-beta
TIM-1	T-cell immunoglobulin mucin receptor 1
TIMP-2	Tissue inhibitor of metalloproteinases-2
TOPCAT	Treatment of Preserved Cardiac Function Heart Failure with an Aldosterone Antagonist Trial
UACR	Urinary albumin to creatinine ratio
uL-FABP	Urinary Liver-type Fatty Acid Binding Protein
USD	United states dollars
WRF	Worsening renal function
β2-M	Beta-2-microglobulin

## Appendix A. Classification of Cardiorenal Syndrome

Cardiorenal syndrome (CRS) encompasses a spectrum of disorders where acute or chronic dysfunction of the heart and kidneys is closely interconnected. To facilitate clinical understanding, CRS is commonly divided into five phenotypes based on the primary affected organ, the time course, and whether a systemic process is involved. The Table A1 below summarizes these subtypes with their key features and representative clinical examples [13,14].

**Table A1:** Classification and mechanisms of cardiorenal syndrome [1,2]. ACRS: Acute cardiorenal syndrome; AHF: Acute heart failure; AKI: Acute kidney injury; CKD: Chronic kidney disease, HF: Heart failure.

Phenotype	Description	Clinical Examples
Type 1—Acute cardiorenal syndrome (ACRS)	Acute heart failure (AHF) resulting in acute kidney injury (AKI)	Acute myocardial infarction, Cardiogenic shock, Acute decompensated heart failure
Type 2—Chronic cardiorenal syndrome	Chronic heart failure progressively causing chronic kidney disease (CKD)	Chronic heart failure of any etiology
Type 3—Acute renocardiac syndrome	Acute kidney injury precipitating acute cardiac dysfunction	Fluid overload, Uremic metabolic disturbances, Inflammatory surge
Type 4—Chronic renocardiac syndrome	Chronic kidney disease driving the development of chronic heart failure	Left ventricular hypertrophy, CKD related cardiomyopathy
Τype 5—Secondary CRS	Systematic disorder simultaneously impairing both heart and kidney	Amyloidosis, Sepsis, Cirrhosis
